# The use of DOIs within government publishing: Benefits and costs

**DOI:** 10.23889/ijpds.v8i2.2210

**Published:** 2023-09-14

**Authors:** Louise Corti

**Affiliations:** 1 Office for National Statistics, London, United Kingdom

## Objectives

Digital Object Identifiers (DOI) are a standard feature within academic publishing, helping make resources findable, citable, connected, and impact-trackable. This talk describes how the UK’s Office of National Statistics has led the way in piloting their use within government and promoted the DOI system as a standard across government publishing.

## Method

In March 2021 a small project was conducted within ONS, led by the Secure Research Service Impact, Data Management and metadata teams, to test a small-scale implementation of DOIs - and their tracking - for its secure data catalogue (https://ons.metadata.works/domain/index.html).

Pilot work commenced with bringing together a cross-ONS working group to appraise and discuss business and user needs for the use of DOIs within the organisation. In 2022, working closely with the British Library, ONS proposed and prepared documentation for the use of the DOI standard as a recommended standard across GOV.UK.

## Results

In late 2021, membership of the British Library DataCite consortium was sought to mint DOIs and implementation was planned with, and undertaken by, the SRS catalogue providers. DOIs were rolled out in early October 2022, representing the first use with UK government, and serving as a valuable pilot to evaluate both user and impact benefits, and implementation costs.

Altmetric was secured as a tool to track mentions of DOIs across the digital landscape - to test out the efficacy of such a system for monitoring use and helping inform impact and evaluation work for data providers and data services.

In spring 2022, the DOI standard was provisionally agreed as a recommended standard across wider UK government by the Open Standards Board and Data Standards Authority.

## Conclusion

This talk provides a valuable reflection and advice on (i) the journey of advocating for/ introducing the concept of DOIs to a government organisation, (ii) the steps required to work this up into a pilot, and (iii) the value of DOIs for impact /evaluation work within a government-led data organisation.

